# Proteolysis of vaginally administered bovine lactoferrin: clearance, inter-subject variability, and implications for clinical dosing

**DOI:** 10.1007/s10534-022-00481-7

**Published:** 2022-12-29

**Authors:** Thomas P. Hopp, Maura-Ann H. Matthews, Klaudyna Spiewak, Zafeiria Athanasiou, Richard S. Blackmore, Gary A. Gelbfish

**Affiliations:** 1 Metrodora Therapeutics LLC, Brooklyn, NY USA; 2 Department of Surgery, Mount Sinai School of Medicine, New York, NY USA

**Keywords:** Bovine lactoferrin, MTbLF, Lactoferricin, Vaginal fluid, Proteases, Bacterial vaginosis

## Abstract

This report describes proteolytic fragmentation and clearance of bovine lactoferrin (bLF) upon intravaginal administration in premenopausal women. Tablet formulations (MTbLF) containing 300 mg of bLF progressed through three phases: Pre-Dissolution, Dissolution, and Washout, over a 30-h time course. Tablets dissolved slowly, replenishing intact 80 kDa bLF in vaginal fluid (VF) as proteolysis occurred. bLF was initially cleaved approximately in half between its N- and C-lobes, then degraded into sub-fragments and small peptides. The extent of proteolysis was less than 10–20% across multiple subjects. Concentrations of both intact 80 kDa bLF and smaller fragments decreased in VF with a similar time course suggesting washout not proteolysis was the main clearance mechanism. Concentrations of intact and/or nicked 80 kDa bLF peaked between 4 and 8 h after administration and remained above 5 mg/mL for approximately 24 h. Experiments with protease inhibitors in ex vivo VF digests suggested an aspartyl protease was at least partially responsible for bLF cleavage. However, digestion with commercial pepsin or in vivo in the human stomach, demonstrated distinctly different patterns of fragments compared to vaginal proteolysis. Furthermore, the 3.1 kDa antimicrobial peptide lactoferricin B was not detected in VF. This suggests pepsin-like aspartyl proteases are not responsible for vaginal proteolysis of bLF.

## Introduction

Bovine lactoferrin (bLF) is a single chain, 80-kDa glycoprotein of the transferrin family consisting of 689 amino acids (Farrell et al. 2004; Baker and Baker 2004) in two homologous, 40 kDa N- and C-terminal lobes, each containing an iron binding site that binds one ferric (Fe^3+^) ion (Moore et al. 1997). Lactoferrin is selectively bactericidal, contributing to the maintenance of healthy microbiomes in the gastrointestinal tract and vagina by sparing lactobacilli, bifidobacteria, and other commensal organisms, while killing pathogens (Oda et al. 2014; Wen et al. 2017; Pino et al. 2017; Valenti et al. 2018). Its bacteriostatic and bactericidal effects result not only from its ability to bind iron but also from short, polycationic sequences that bind directly to microbial cell surfaces (Dionysius and Milne 1997; Tomita et al. 1991; Kuwata et al. 1998; Hwang et al. 1998; Haney et al. 2007; Bellamy et al. 1993).

The therapeutic potential of orally administered bLF has been extensively explored in various indications (Superti 2020) and proteolysis of orally dosed bLF has been well characterized (Baker and Baker 2004; Rastogi et al. 2014a, b; Troost et al. 2001; Furlund et al. 2013). The use of vaginally administered bLF remains an emerging field with potential for therapeutic benefit based on its anti-inflammatory properties as well as its selective antimicrobial activity (Artym and Zimecki 2021; Valenti et al. 2018). We and others postulate that depletion of iron from the vaginal environment with bLF will have beneficial effects on the vaginal microbiome since lactobacilli, the bacteria that dominate a healthy microbiome, do not require iron for growth (Archibald 1986) while bacteria common to vaginal infections, including bacterial vaginosis (BV), require iron (Jarosik et al. 1988). The therapeutic challenge is to develop vaginal formulations of bLF that deliver sustained iron-binding capacity, as well as to understand the relative importance of vaginal proteolysis and antimicrobial peptides.

The safety, vaginal pharmacokinetics, and proteolysis of different formulations of Metrodora Therapeutics bovine Lactoferrin (MTbLF) is under evaluation in a Phase 1 clinical trial enrolling healthy women and women with BV (ANZCTR registration number ACTRN 12619000295145). While complete pharmacokinetic results are not yet available from that study, samples of vaginal fluid (VF) from a subset of patients enrolled and formulations tested have been used to characterize proteolysis and clearance of vaginally administered bLF. We recently reported the sequences of vaginal proteolysis products using a combination of western blotting, RP-HPLC fractionation, and peptide mass spectrometry (Hopp et al. 2022). Herein, we extend those findings by examining the extent and variability of proteolysis as well as its overall contribution to clearance of vaginally administered bLF. We also compare gastric fluid and peptic fragmentation patterns to those observed in our clinical samples and conclude that proteolysis in VF is clearly different from stomach proteolysis despite the acidic nature of both fluids.

The predominant vaginal cleavage site for bLF is Tyr 324 yielding a 43 kDa C-lobe fragment and a 37 kDa N-lobe fragment, both of which retain intact iron-binding sequences (Hopp et al. 2022). Other fragment pairs observed included 58 + 22 kDa, 18 + 62 kDa, and 16 + 64 kDa bands with the smaller component from the N-lobe and the larger from the C-lobe. As we reported previously, it is important to assess fragmentation using both reducing and non-reducing gels, because bLF contains 17 disulfide bonds and some nicked species appear as less than 80 kDa on reducing gels but are likely still 80 kDa in vivo. Our previous report used a novel visualization method for bLF fragmentation, a tri-color western in which the results of western blotting with anti-bLF N-lobe and C-lobe monoclonal antibodies (mAbs) were shown in red and green, respectively, superimposed on PAGE gels of the same specimens stained with Coomassie blue (Hopp et al. 2022). That same red, green, blue color convention is utilized in this report, where additional time-course PAGE gels are provided in order to follow the extent and variations of proteolysis.

## Materials and methods

### MTbLF

Vaginal proteolysis experiments (clinical trial specimens and ex vivo digests) used MTbLF, which is a high-purity product isolated from pasteurized skim cows’ milk by cation exchange chromatography and ultrafiltration in cGMP-compliant facilities. The MTbLF was used as freeze-dried powder (ex vivo digests) or formulated as tablets for vaginal administration. Suppository tables were compacted from blended solid components including 300 mg of MTbLF, filler, binder, and various gelling and mucoadhesive agents. MTbLF is predominantly the bLF-b form, noted in the literature as being unglycosylated at asparagine 281, while the bLF-a form is highly glycosylated at that position (Wei et al. 2000). Asparagines 233, 368, 476, and 545 in MTbLF are highly glycosylated with carbohydrate moieties averaging 1,080 Daltons each (Hopp et al. 2022). These carbohydrate moiety weights were used in calculating theoretical molecular weights of bLF fragments in polyacrylamide gel electrophoresis (PAGE) experiments. In vitro pepsin digests were performed with MTbLF. Gastric digestion experiments were performed with food grade bLF (Natraferrin from Murray Goulburn Co-Operative Co. Ltd, Leongatha, Australia).

### Vaginal fluid sample collection and storage

VF specimens were collected before and after vaginal administration of single 300 mg MTbLF tablets, inserted near the cervix with a plastic applicator. At various time points after the dose was inserted, PVA Lasik Spears (eye surgery absorbent sponges) were used to retrieve fluid from the vagina. Swabs were frozen at −20 °C until processed. VF was isolated from thawed swabs by centrifugation through Costar 0.45 µm Spin-X filters. VF was diluted with 4× sample buffer for SDS-PAGE. Premenopausal women between the ages of 18 and 50 who agreed to abstain from sexual intercourse for at least 24 h prior to dose administration were eligible to participate in the study.

### Ethics approval and regulatory clearance

The trial in which the clinical specimens were collected had approval from the Human Research and Ethics Committee of the Alfred Hospital, Melbourne, Australia (Project number 493/18). All participants gave written informed consent. In compliance with Australian regulations, Metrodora Therapeutics, the sponsor, notified Australia’s Therapeutic Goods Administration (TGA) via the Clinical Trial Notification scheme (CTN; clinical trial: CT-2018-CTN-03362-1) and registered with the Australian New Zealand Clinical Trials Registry (ANZCTR; ACTRN12619000295145) prior to starting the trial.

### bLF pepsinization and gastric digestion experiments

In vitro digestion of MTbLF was carried out with porcine pepsin obtained from Sigma Aldrich (catalogue #P6887). Digests were performed by Tomita’s method (Tomita et al. 1991), dissolving 250 mg of MTbLF in 5 mL of water (50 mg/mL), adjusting the pH to 3.0 with 1 N HCl, adding 7.5 mg solid pepsin (3% w/w), and incubating at 37 °C. Samples were withdrawn at various time points (including a zero-time sample taken before adding the enzyme). A heavy white precipitate formed and remained for the duration of the procedure. Digest aliquots were taken at 15, 30, 60, and 240 min after adding the enzyme, and the reaction stopped by mixing with a four-fold excess of SDS sample reducing buffer (Invitrogen). Samples were stored frozen until utilized in PAGE and western blot analyses.

Gastric digestion experiments were conducted using an 8 mg/mL solution of food grade bLF in water in a single-subject research experiment. Samples were collected with informed consent from the fasted individual (GAG, self-experimentation) via an orogastric catheter placed in the stomach. Gastric fluid (2.5 mL per timepoint) was withdrawn prior to oral ingestion of 250 mL of bLF, and at various times after ingestion. Samples were collected into 15 mL plastic centrifuge tubes and digestive activity was stopped in an aliquot from each sample by mixing with SDS PAGE reducing buffer and heating at 90 °C for 10 min.

### Polyacrylamide gel electrophoresis

PAGE analysis used the Invitrogen NuPAGE system with precast 4–12% acrylamide gradient gels and Coomassie brilliant blue or western staining. Clinical samples already in PAGE buffer were diluted tenfold with sample buffer for loading on PAGE gels. SeeBlue-Plus-2 pre-stained standard protein ladders (Invitrogen) were used in PAGE and western experiments. Standard proteins were run on all gels, including standard MTbLF, SeeBlue-Plus-2 standards, or both, so that each gel had at least one standard lane and sometimes several. These standard lanes were used to superimpose gel images to precisely compare fragment sizes. This was especially critical for comparing fragments in multiple subjects’ VFs or separate doses to the same subject. PAGE gels were scanned with an Epson Perfection V700 Photo Scanner, Model J221A. Band intensities were measured with Epson Scan software version 3.9.2.1US and integrated band volumes were reported with CLIQS software version 1.1.

### Western blots

PAGE gels were transferred to nitrocellulose filters using Invitrogen’s iBlot system. Color was developed with Invitrogen’s Western-Breeze reagents, with one exception: the kit’s milk-based blocking solution was replaced with 1% w/v bovine serum albumin dissolved in wash buffer, to avoid interference from bLF present in milk products. Alkaline phosphatase-conjugated goat-anti-mouse secondary antibodies and chromogenic substrate were supplied in the kit. Primary antibodies were murine N-lobe-reactive anti-bovine lactoferricin B mAb (GWB-C768F7) and anti-bLF C-lobe mAb (GWB-1A2A49) obtained from Genway.

### Reversed-phase HPLC analysis

Reversed-phase HPLC was used to separate and analyze bLF and its VF fragments (Hopp et al. 2022). A Hewlett Packard 1050 HPLC system with a C3 reversed-phase column (RPC3; Zorbax 300SB-C3, 3.5 µm, 4.6 × 150 mm, Agilent #863973-909) equipped with a guard column (Zorbax 300SB-C3, 5 µm, 4.6 × 12.5 mm (Agilent #820950-924) was operated at 25 °C. Solvent A was 0.1% trifluoroacetic acid in water; solvent B was 0.1% trifluoroacetic acid in acetonitrile. A gradient from 10 to 70% B in 17 min at 1.0 mL/min separated bLF and its major fragments. Clinical VF samples were passed through Spin-X 0.45 µm centrifugal filters before injection onto the HPLC.

### Ex vivo digestion of MTbLF in vaginal fluid

Vaginal fluid for ex vivo digests was purchased from Lee Biosolutions, Maryland Heights, MO (catalogue #991-10-S). The VF specimens had been stored at −20 °C without additives.

MTbLF was digested in ex vivo conditions as follows: the powdered MTbLF was dissolved at 20 mg/mL in 100 mM lactic acid buffer pH 3.3, then combined with the VF specimen, which had been pH-adjusted to 3.3 with HCl and filtered through a 0.45 µm Spin-X filter. Digestion positive controls were carried out, as well as protease inhibitor experiments with pepstatin and EDTA, as follows:

#### Acidified pepstatin solution

Pepstatin was first dissolved at 1 mg/mL in DMSO and then diluted with 100 mM lactic acid buffer pH 3.3, to make a 4 µg/mL inhibitor solution. When combined 1:4 with other ingredients, as in Table 1, it yielded a 1 µg/mL final concentration of pepstatin.

**Table 1:** VF + bLF + inhibitor mixtures

Mixture	VF (µL)	Inhibitor (µL)	100 mM lactic acid pH 3.3 (µL)	Acidified MTbLF (µL)
Control digest	100	–	50	50
+ Pepstatin	100	50	–	50
+ EDTA	100	50	–	50

#### Acidified EDTA solution

EDTA was dissolved at 40 mM in 100 mM lactic acid pH 3.3, then combined 1:4 with other ingredients (Table 1) to yield a 10 mM final concentration of EDTA.

The digestions and inhibition experiments were carried out as follows: (1) aliquots of acidified VF stock were first combined with inhibitor stocks as indicated in the second and third columns of Table 1. (2) For the positive digestion control, 100 mM lactic acid, pH 3.3 was substituted for the inhibitor solutions to balance the volumes with inhibitor samples (fourth column). (3) All samples were mixed and chilled on ice. (4) 50 µL of iced acidified MTbLF was added to all three samples to achieve the final reaction mixtures without starting the digestion. (5) Zero time point samples were taken from all samples, 10 µL aliquots combined with 90 µL of 1.11× SDS blue sample reducing buffer and heated at 90 °C for 10 min to prevent the reaction. (6) Reaction mixtures were incubated in a 37 °C water bath to commence the digestion reactions. (7) Time-point aliquots of 10 µL were removed at 2 h, 4 h, and 24 h, combined with 90 µL of 1.11× SDS buffer and heated at 90 °C for 10 min.

## Results

### Sustained release of MTbLF and persistence of fragments in vaginal fluid

**Fig. 1:**
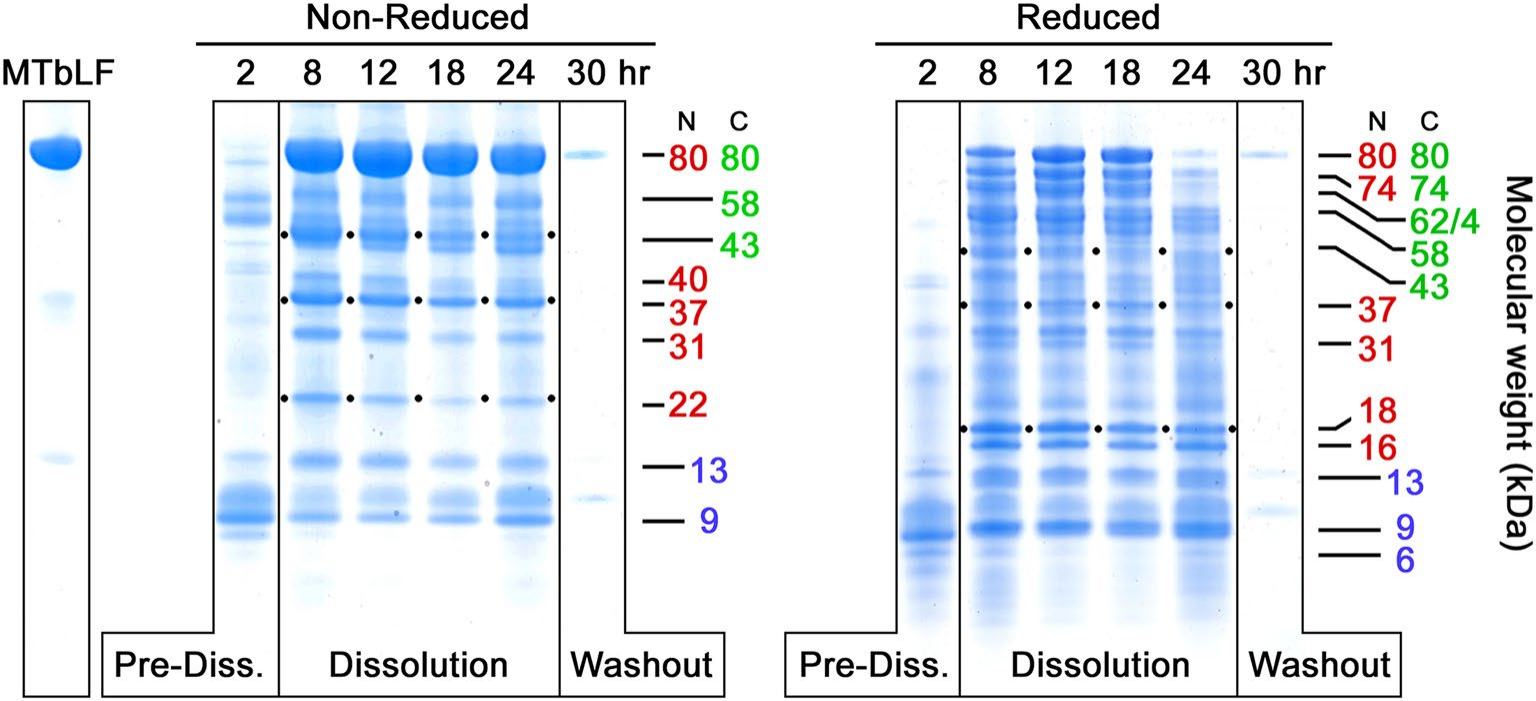
Time course of VF samples from Subject 104, Formulation 2. Two PAGE gels are shown for the same subject. The left-hand gel shows the time course series of VFs with disulfide bonds intact. The right-hand gel shows the same samples after reduction to break disulfide bonds. To the right of each series, the molecular weights of the main fragments are shown, color coded with their location in the N lobe (red) or C lobe (green) as was previously determined (Hopp et al. 2022). Black dots within the Dissolution phase are included to aid the eye in following four bands of interest, 43 kDa, 37 kDa, and either 22 kDa (lefthand PAGE) or 18 kDa (righthand PAGE). Several lanes shown in this figure (lanes 4, 6, 8, 10, and 12 from left) appeared in our previous publication (Hopp et al. 2022), while most have not been published before

**Fig. 2:**
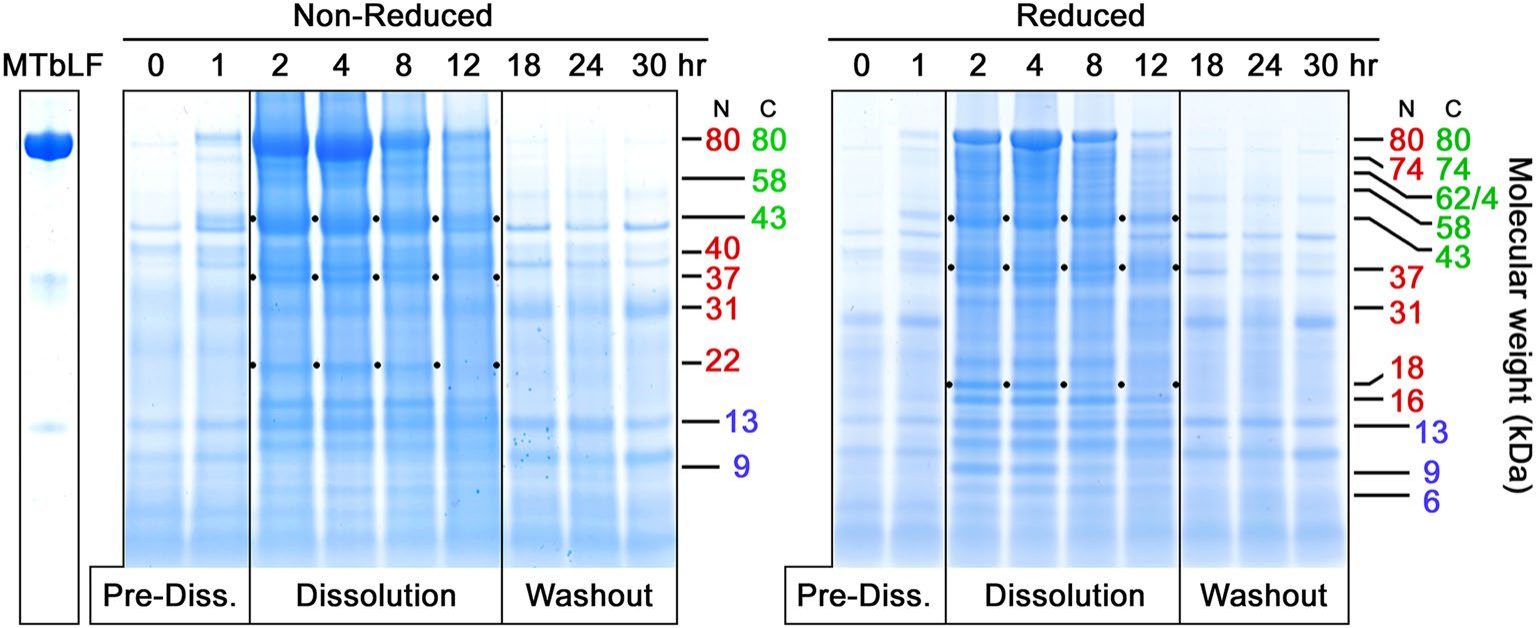
Time course of VF samples from Subject 103, Formulation 1. Layout and labeling are the same as for Fig. 1. Note that the molecular weight labels relate to the central Dissolution phase, and do not match the Washout phase of normal VF protein bands. The black dots assist in following several selected bands, as in Fig. 1. Compared to Fig. 1, these gels show more rapid dissolution of the dose into VF, but an equally abrupt Washout phase. Nicking is apparent in the relatively lesser amounts of 80 kDa bLF and 43 kDa fragment in the reduced, vs non-reduced gel

**Fig. 3:**
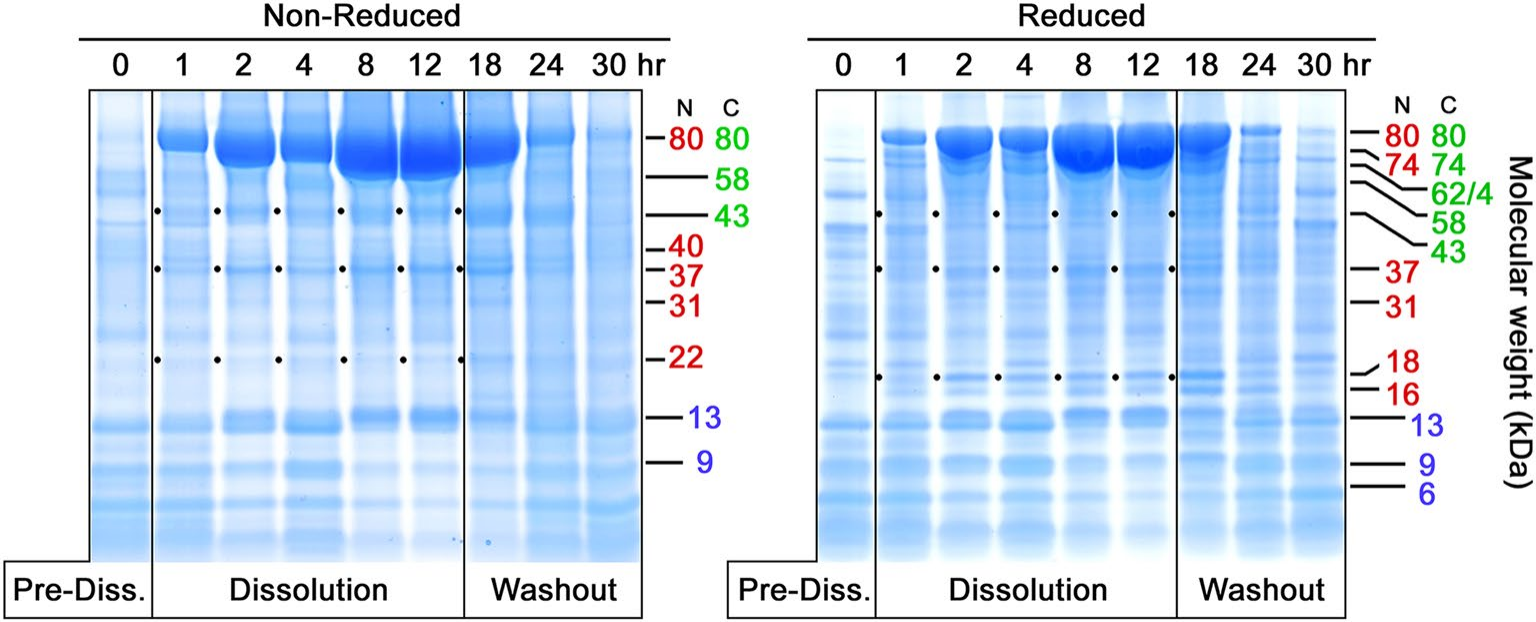
Time course of VF samples from Subject 102, Formulation 2. Layout and labeling are the same as for Figs. 1 and 2. Notable are much darker bands for 80 kDa bLF on both the non-reduced and reduced gels, suggesting most of the 80 kDa material was neither cleaved nor nicked by vaginal proteases in this subject

**Fig. 4:**
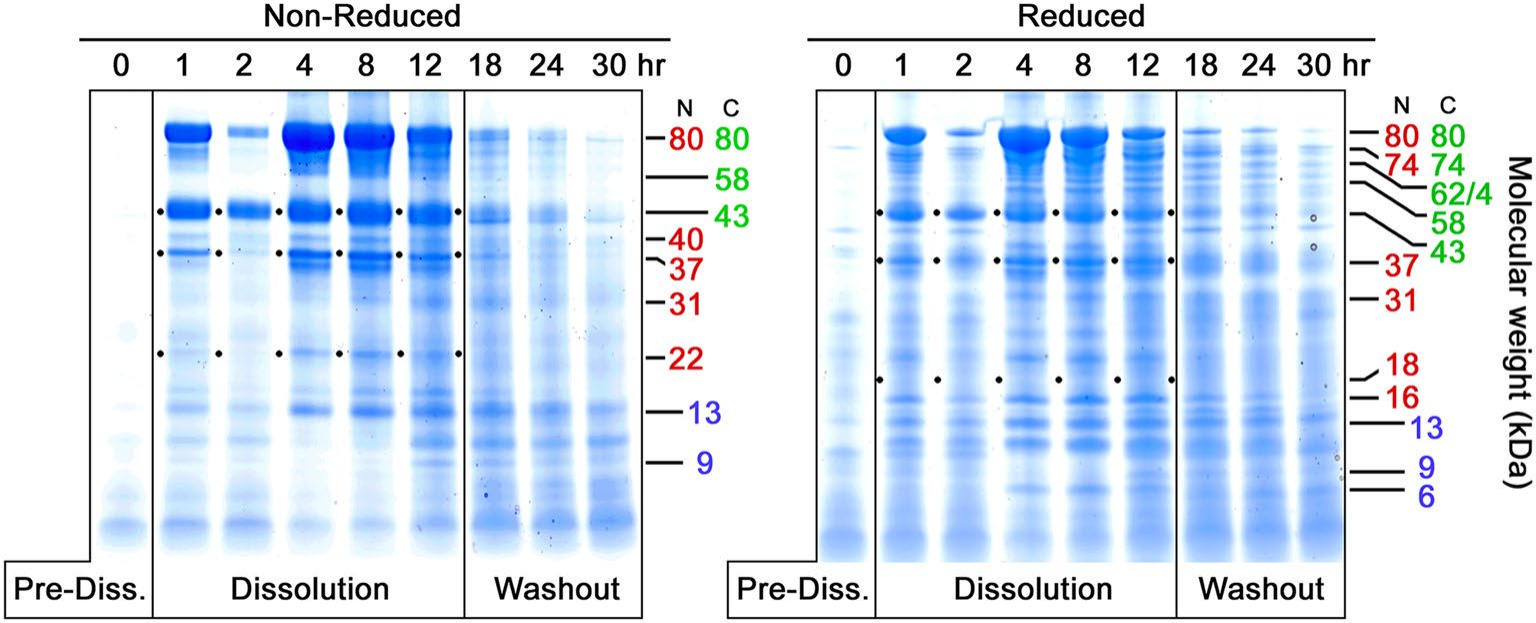
Time course of VF samples from Subject 103, Formulation 3. Layout and labeling are the same as for Figs. 1, 2, and 3. Notable are the intermediate level of proteolysis of 80 kDa bLF and substantial amounts of 43- and 37-kDa fragments on both the non-reduced and reduced gels, suggesting a relatively low level of nicking in these samples. Also notable are the lack of the 18 kDa fragment band on the reduced gel, as well as a moderate precursor/product diagonal. The diminished protein levels in the 2 h lanes illustrates inhomogeneity of bLF distribution early in the Dissolution phase

Figures 1, 2, 3, and 4 show reduced, and non-reduced SDS-PAGE analysis of VF specimens collected pre-dose and over a 30-h time course after vaginal administration of a solid dose tablet containing 300 mg MTbLF. To facilitate comparison, all samples were loaded at the same volume and dilution. VF was diluted with PAGE sample loading buffer such that 0.25 µL of VF was loaded in all sample wells. This allowed direct comparison of all electrophoresed samples. Visual inspection was sufficient to estimate the relative amounts of bLF and fragments from lane to lane, and gel to gel. However, all lanes of all gels were also scanned using an Epson gel scanner. These scans allowed quantitation of bLF and fragments and confirmed the accuracy of visual comparisons. The small VF sample volume (0.25 µL) was used because while pre-dose VF specimens contained 10–20 mg/mL protein spread across multiple bands, the concentration of 80 kDa bLF in many dosed samples was > 50 mg/mL. It should be noted that VF contains human lactoferrin (hLF) at low and variable levels (Roberts et al. 2019; Cohen et al. 1987) on the order of micrograms per milliliter. The multi-milligram per milliliter levels of MTbLF overwhelm any potential hLF bands that might otherwise be visible with Coomassie blue staining. In addition, none of our western gels, which were probed with bLF-specific mAbs, showed any detectable levels of hLF or its fragments (data presented herein and Hopp et al. 2022).

In Fig. 1, many lower molecular weight bLF fragments are seen in VF samples compared to the intact MTbLF lactoferrin (at left). In this figure and three subsequent examples, the lanes are labeled at the top with the timepoint at which the VF sample was taken. Lanes are grouped in boxes according to the pharmacokinetic progression of the MTbLF tablet formulation through three recognizable phases: a Pre-Dissolution phase, in which only pre-existing VF proteins appear in the lane(s), a Dissolution phase, during which the tablet dissolves and reaches high concentrations of bLF in VF (and fragments when/if proteolyzed), and a final Washout phase during which the lanes return to the pre-existing VF protein pattern or, as in this case, drop to a level of VF proteins lower than pre-dissolution. The term washout is used to describe what appears to be intact bLF and bLF fragments being cleared as part of normal VF turnover.

While a few background bands pre-exist in the Pre-Dissolution phase VF at this level of loading (0.25 µL VF/lane), almost all dark bands in other lanes are 80 kDa MTbLF or proteolytic breakdown products of bLF.

Figures 1, 2, 3, and 4 each show a pair of non-reduced vs reduced PAGE gels, each with matched VF samples from a single subject, differing only in the oxidation–reduction state of their disulfide bonds. While the in vivo state of bLF is likely oxidized as BV is associated with decreased redox potential in VF (Holmes et al. 1985), analyzing VF samples using both non-reducing and reducing gels provides additional insight into the extent of proteolysis that occurs in vivo. In some instances, including Fig. 1, the differences between reduced and non-reduced samples are dramatic. While inspection of the left-hand gel in Fig. 1 makes it clear that a very large amount of bLF was released from the tablet by the 8-h time point and persisted to 24 h before washout by 30 h, this bLF was not fully intact. The right-hand PAGE shows that a large proportion of the 80 kDa material was proteolytically nicked and held together by disulfide bonds, leading to many lower molecular weight bands on the right-hand, reduced gel. This nicking was so substantial that by the 24 h time point virtually all of the 80 kDa bLF had been nicked at least once, if not multiple times, leaving only trace amounts of full length 80 kDa bLF in that lane.

#### Precursor-product diagonals

In a closed system, proteolytic degradation typically shows a precursor-product relationship with fragments of lower molecular weight accumulating as higher molecular weight molecules are clipped into smaller pieces. This trend is only faintly present in Fig. 1, and only noticeable in the reduced lanes at 24 h. Instead, the fragment pattern appears stable over time, suggesting that intact 80 kDa bLF was replenished throughout most of the time course as the solid MTbLF tablet dissolved, releasing additional bLF into the vaginal space, which in turn provided a continuous source of 80 kDa material, thereby replenishing the fragments for up to 24 h. Subsequently, all bLF-related bands disappeared simultaneously, suggesting that once the dose was completely dissolved, the natural flushing action of VF carried away any remaining intact bLF along with its fragments. None of the VF samples in this study demonstrated a clearcut diagonal precursor/product pattern, suggesting proteolysis does not play a major role in clearance of bLF from VF, compared to washout.

Figure 2 shows two PAGE gels of VF samples collected from a second subject that also received 300 mg intravaginal MTbLF. These gels again show evidence of nicking in that the bands for both 80 kDa bLF and the 43 kDa fragment are stronger on the left and much diminished on the right after disulfide reduction. Seen here again is the parallel occurrence of intact bLF and many of its fragments, again implying slow delivery of 80 kDa material from the tablet combined with rapid nicking of bLF to smaller fragments. Of additional interest in these images is evidence of a much different time-course of dose dissolution and washout. In Fig. 1, the dose had not dissolved by 2 h, and only reached high levels of bLF between 8 and 24 h. On the other hand, in Fig. 2, the dose had substantially dissolved by 2 h, showed evidence of washout by 12 h, and was gone by 18 h. The more rapid tablet dissolution in this case may be attributable to inter-subject variation, minor formulation differences, or both. A larger subject population will be required to make a definitive determination.

#### Low proteolysis in some individuals

Figure 3 shows PAGE results for another clinical subject who received the same formulation as the subject shown in Fig. 1. Once again there was a rapid dose release into VF, with impressively larger amounts of 80 kDa bLF and lesser amounts of 43 and 37 kDa fragments, suggesting proteolysis was low in this subject. Proteolysis was present, however, and its main products were the 43 and 37 kDa bands, visible in the lefthand PAGE. Moderate amounts of nicking can be inferred from the diminished presence of the 43 and 37 kDa bands in the righthand, reduced PAGE. The presence of large amounts of 80 kDa bLF appeared to result in a long, trailing Washout phase extending to nearly 24 h. Although fragment bands are much less prominent than on most PAGE gels, they are nevertheless the same bands seen in other subjects.

Figure 4 shows a subject with intermediate levels of proteolysis. While the 80 kDa bands are significantly reduced and fragment bands are considerably darker than seen in Fig. 3, there is nevertheless a continuous phase in which intact bLF is present, from 1 h post dosing through 12 h with lesser amounts of intact bLF continuing through a long, tailing Washout phase to 30 h post dose.

In Fig. 4 there are several notable phenomena, which have been occasionally observed on other PAGE gels not shown here. The 2 h time point shows less bLF than the 1 h time point. Given that such variations usually occur in the early timeframe after dosing, we interpret this as inhomogeneous distribution of the dose within the vaginal space as it first begins to dissolve. Later samples show more consistent levels of bLF once it has had time to disperse.

#### 18 kDa fragment

Another unusual finding with the VFs shown in Fig. 4 is a complete lack of the 18 kDa fragment on the right-hand, reduced gel. Given that most reduced gels of samples from other subjects show a strong 16 + 18 kDa band pair, the lack of any 18 kDa is notable. As will be discussed below, this lack of the 18 kDa band could hypothetically relate to the absence of a responsible protease, or altered conditions in these VF samples (pH, ionic strength). Here it is worth noting that the absence does not appear to be an isolated artifact of electrophoresis, given that it is uniformly absent across all lanes, while its 16 kDa counterpart is present consistently in almost all lanes, paralleling higher molecular weight bands from which it is derived by nicking.

Finally, this gel pair, especially the non-reduced gel, shows a moderate upper-left, lower-right diagonal reflecting the precursor/product pattern of proteolytic fragments mentioned above. The trend is not strong but implies that in this particular subject, neither washout nor proteolysis can fully explain the band patterns. Instead, it indicates that both mechanisms may play parts in the disappearance of dosed bLF from VF, albeit to different extents.

### HPLC patterns of MTbLF fragments

**Fig. 5:**
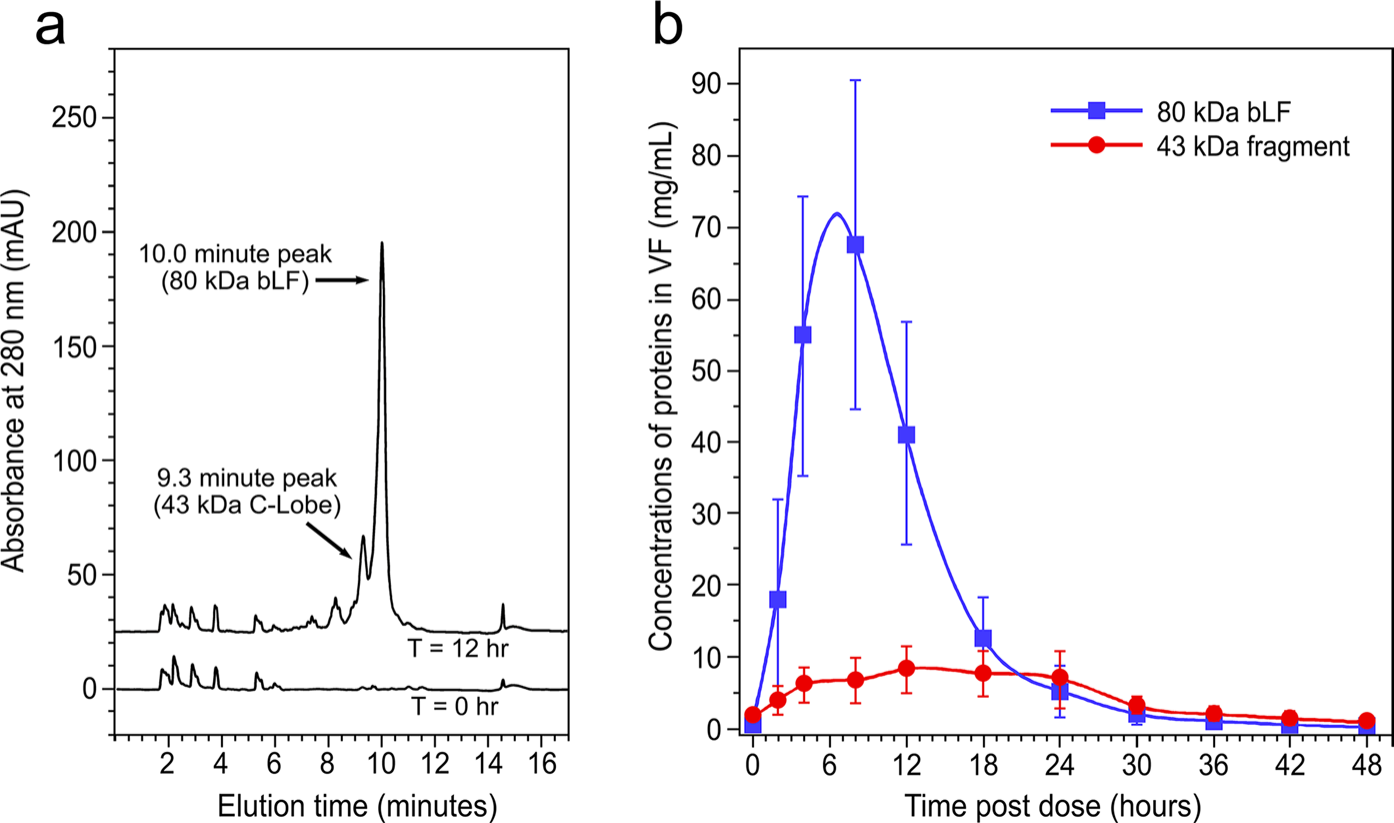
RPC3 HPLC separation of clinical VF samples. **a** Shows absorbance traces for T = 0 and T = 12 h samples from a single subject that received 300 mg MTbLF as a vaginal tablet. The 10-min peak was confirmed in separate calibration runs as the normal elution time for intact bLF. The prominent proteolysis product at 9.3 min represents the 43-kDa C-lobe fragment. **b** Shows the concentrations of 80 kDa bLF and the 43 kDa fragment expressed as mg/mL in VF from 18 dose administrations (n = 6 subjects, 3 dose administrations each). The median age of the women was 26 (range 23–38). Bars represent the 95% confidence interval of the mean concentration at each timepoint; data were fit with a soft SPLINE

While PAGE and western blot methods provide much useful detail about the number and size of proteolytic fragments, HPLC represents a high-throughput method to monitor amounts of intact vs proteolyzed MTbLF in large numbers of clinical VF samples. Figure 5a shows the RPC3 reversed phase HPLC separation of two samples from the time series of one woman on a single day of dosing. The T = 0 h plot shows the profile of VF proteins prior to dosing, while the T = 12 h plot shows the pattern of peaks near the time of maximum bLF concentration. The intact bLF molecule elutes in a large peak at 10 min while the 43 kDa N-lobe fragment appears at 9.3 min. The nicked 80 and 43 kDa materials were determined to run in the same peaks as their intact counterparts (Hopp et al. 2022). The 37 kDa N-lobe fragment usually comprised a small amount of material that eluted in the tailing region of the 10-min peak without giving a distinct peak.

Figure 5b shows the mean concentration–time profile assembled from 6 different women that each received three 300 mg doses of MTbLF (n = 18 dose administrations) with each dose administration separated by 48 h. The large confidence intervals reflect variability in the maximal concentration of 80 kDa bLF and time variations of the Pre-Dissolution, Dissolution, and Washout phases across 6 subjects. Mean peak concentrations of 80 kDa bLF were ~ 70 mg/mL while levels of the 43 kDa fragment were substantially less, reaching between 5 and 10 mg/mL over the 48 h time course. The level of 43 kDa material was approximately 10% of the 80 kDa material for most of the time course but persisted longer as the 80 kDa material decreased. Late in the time course, the 43 kDa material predominated over the 80 kDa material. Subsequently, both peaks tapered off together over time. The results demonstrate proteolysis is not a major clearance mechanism for MTbLF.

### PAGE and western comparisons with pepsin and stomach fluid digests

**Fig. 6:**
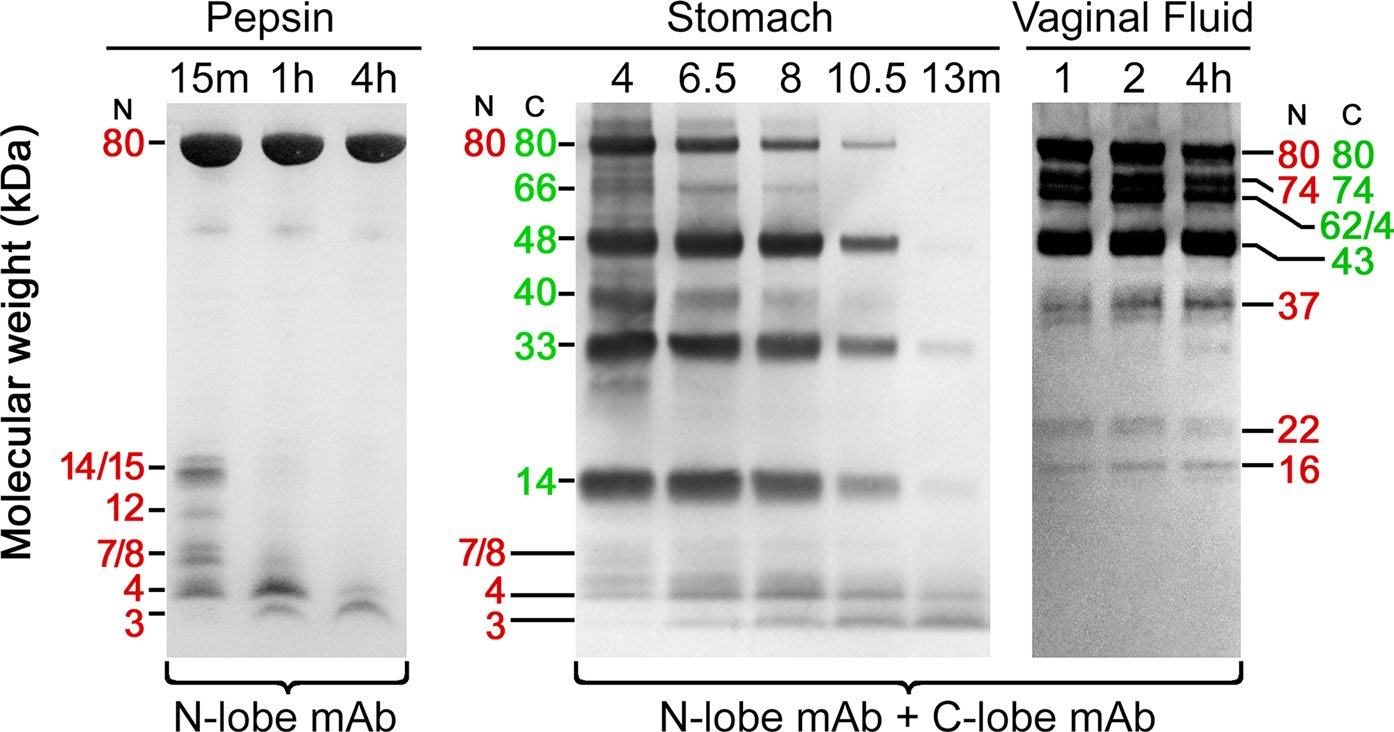
Pepsin and gastric digestion of MTbLF compared to ex vivo VF digestion. Western blots reveal anti N- and anti C-lobe mAb-reactive fragments. As in Figs. 1, 2, 3, and 4, red numbers indicate bands reacting with anti-N-lobe mAb while green numbers indication reaction with anti-C-lobe mAb. Lefthand western shows a partial digest of MTbLF in vitro, using commercially available porcine pepsin and reacted with anti-N-lobe mAb only. In the middle western, stomach aspirates after oral ingestion of food grade bLF were developed with a mixture of the anti-N-lobe and anti-C-lobe mAbs. The righthand western shows MTbLF incubated ex vivo in VF and developed with both mAbs. All samples had disulfide bonds reduced

The identity of the VF protease or proteases that cleave bLF remains to be fully elucidated. Because VF is an acid medium (pH ~ 4), we anticipated the responsible protease(s) might be members of the acid proteinase (aspartyl protease) family that includes pepsin and several homologous acid-active proteases. Figure 6 shows western blots comparing the fragmentation patterns from pepsin or stomach fluid to the pattern observed in VF. There are very substantial differences in the bLF fragments produced by gastric and vaginal enzymes. Most notably, pepsin (left) shows none of the bLF N-lobe mAb-reactive species commonly observed in vaginally proteolyzed bLF. Absent are the 74, 40, 37, 35, 22, 18, and 16 kDa N-lobe reactive fragments, while a different set of fragments appear in the low molecular weight region, including 15, 12, 8, 7, 4, and 3 kDa species, the last of which corresponds with the antimicrobial peptide lactoferricin B (Tomita et al. 1991). The absence of all larger fragments likely results from rapid, complete peptic cleavage at a site near the N-terminus of bLF that separates the 15 kDa N-terminus from the rest of bLF. Following this rapid cleavage, only smaller peptides can be derived from the 15 kDa material by pepsin.

The western blot of gastric aspirates (center) demonstrates that a variety of C-lobe reactive fragments are produced in stomach fluid following oral administration of bLF, including 66, 48, 33, and 14 kDa species. Also clearly visible in this image are the anti-N-lobe bLF mAb reactive fragments of 8, 7, 4, and 3, the last of which is, again, lactoferricin B.

A western blot of VF (right) was included for comparison. It possesses all the major antibody-reactive bands noted in Figs. 1, 2, 3, 4 above. These form a fragment pattern that is distinct from the western blots of ex vivo pepsin digests and gastric aspirates, implying that the protease(s) involved must differ from pepsin or stomach fluid, which contains pepsin as well as small amounts of gastricsin, a closely related pepsin family member with a virtually identical substrate specificity.

Most striking in these results is the presence and stability of lactoferricin B as a prominent product of both in vitro pepsin digestion and in vivo stomach aspirates, while it is entirely absent in the VF western. This confirms and extends our previous observation (Hopp et al. 2022) that lactoferricin B has not been detected in VF, either in samples collected post MTbLF administration in clinical trials or in the closed system ex vivo digests using VF spiked with MTbLF.

It should be noted here that while the bLF used in these experiments came from multiple sources, we have found that the molecule is highly stable in a variety of forms (powder, tablet, liquid solutions, and a variety of formulations with different excipients). bLF is also stable over a wide pH range as long as no protease is added. Therefore, it is unlikely that these band patterns differ based on differences in the proteolytic susceptibility of bLF from different sources.

### Protease inhibitors

**Fig. 7:**
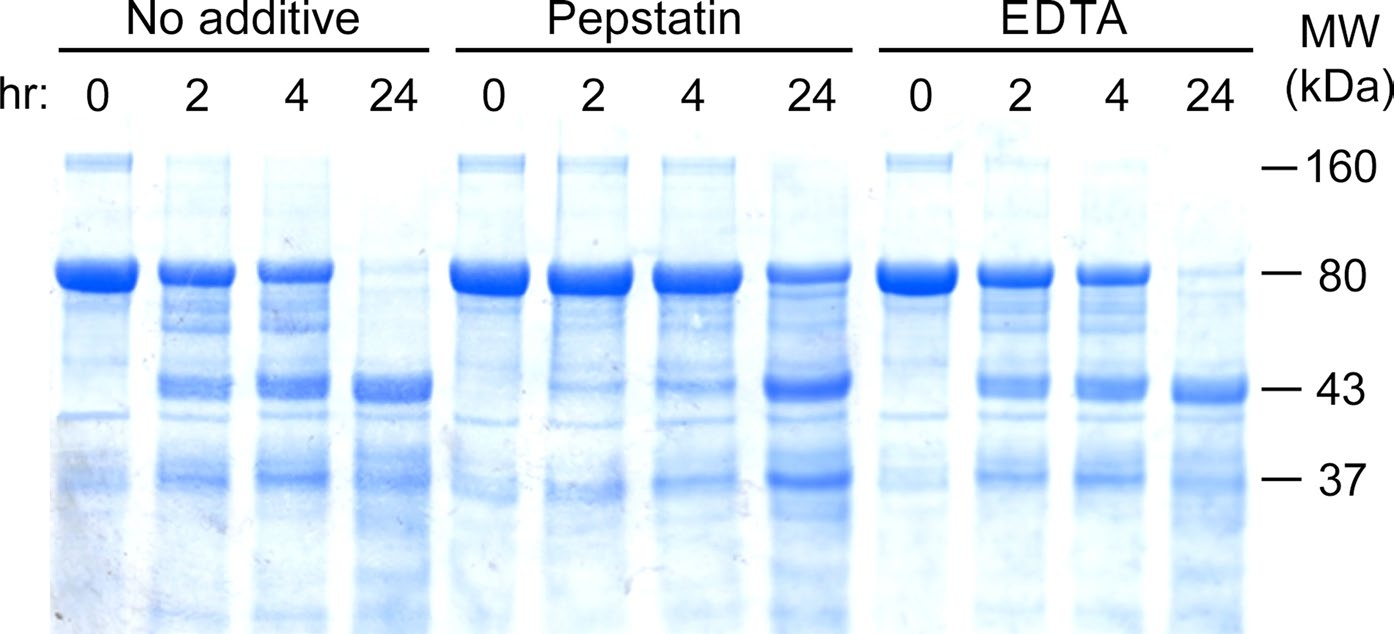
PAGE gel of ex vivo digests of bLF by VF with and without protease inhibitors. MTbLF was incubated at 37 °C with a specimen-bank sample of VF for the times indicated. Each set of four timepoints was treated differently: on the left, no inhibitor was added; at center, pepstatin was added at 1 µg/mL; at right, EDTA was added at 10 mM. Proteolysis was stopped at the time points indicated by mixing with SDS PAGE sample buffer and heating to 90 °C

Given that proteolysis of vaginally delivered bLF is distinct from gastric fragmentation of orally dosed bLF, we wished to learn more about the specific enzyme(s) involved. Figure 7 shows the results of adding protease inhibitors to an ex vivo digest of MTbLF using bLF-naïve VF obtained from a specimen bank. In the lefthand four lanes, a time course of digestion over a 24 h period shows the disappearance of 80 kDa bLF and appearance of the major fragments of 43 and 37 kDa in a typical precursor-product diagonal. The destruction of the 80 kDa protein was essentially complete by 24 h, while the bands at 43 and 37 kDa reached maximum intensity in that time. When the protease inhibitor pepstatin was added to the VF (center four lanes) proteolysis of the 80 kDa bLF was greatly diminished, though not eliminated entirely. Additionally, the bands of 43 and 37 kDa material were darker at the 24-h time point, suggesting that their sub-fragmentation was also inhibited by pepstatin.

In contrast, addition of 10 mM EDTA to the VF had no effect on proteolysis. The destruction of 80 kDa bLF followed an identical time course when comparing the righthand four lanes to the lefthand four. This lack of effect rules out proteinases that utilize catalytic metal ions or are activated by metal ions. Additionally, it is worth bearing in mind that the digestions were carried out at pH 3.3, which further serves to rule out whole classes of proteases that are inactive in the acid pH range.

## Discussion

Vaginally administered MTbLF dosed at 300 mg per tablet passes through three phases: a Pre-Dissolution phase, a Dissolution phase, and a Washout phase. These phases relate to the release of bLF from the tablet as it dissolves, diffusion away from the suppository into VF, and subsequent clearance of bLF from the vagina along with its fragments.

### Pre-Dissolution phase

This initial phase is simply the time after insertion of the suppository, during which MTbLF protein (or its fragments) are not yet detectable in VF. This phase is usually brief, 0–2 h, and variable. The variability observed in this phase may be due in part to hydration status of the subject, slow wetting of the tablet, and/or poor mixing of low concentrations within the irregularities of the vaginal space. This variability tends to even out as the dose continues to dissolve and larger amounts of bLF are distributed more ubiquitously within the vaginal space.

### Dissolution phase

The central portion of each time series corresponds to the interval during which the tablet continues to dissolve, releasing substantial amounts of intact bLF into the vaginal space. Because of the slow-release nature of the suppositories, some intact 80 kDa bLF persists between 4 and 24 h post dose despite the proteolytic activity of VF, and despite ongoing elimination.

### Washout phase

As the tablets become fully dissolved, they cease to be a source of fresh MTbLF, whereupon both bLF and fragments begin to disappear from VF. At this point, the rate of disappearance is nearly simultaneous for both the 80 kDa whole molecule and any fragments that have formed, though a minor tailing of the fragments can sometimes be seen. This strongly suggests that the proteolysis is by no means a complete process, and that fragments and bLF alike are cleared by washout rather than further proteolytic degradation. Our assessment that the protein is washed out or discharged rather than absorbed is based on the known movement of VF from the cervix downward to the introitus. Furthermore, this is consistent with findings of others who estimated VF turnover as approximately 6 mL per day with 0.5–0.75 mL present at any given time (Caramella et al. 2015). However, to characterize other potential mechanisms of elimination beyond the 10–20% proteolysis that we have demonstrated would require additional study. This would include determining if significant amounts of 80 kDa bLF are absorbed via the vaginal mucosa and if bLF is measurable in the blood stream.

### Implications for clinical dosing

Figure 5b shows the mean VF concentrations of bLF and fragments over 48 h after administration of 300 mg of MTbLF. Concentrations of intact and/or nicked 80 kDa bLF peaked between 4 and 8 h after administration and remained above 5 mg/mL for approximately 24 h. This result supports once daily administration of MTbLF because substantial iron-binding capacity persists for that duration. Moreover, our recent in vitro experiments (Pino et al. 2022) have shown that MTbLF has antimicrobial activity against clinical isolates of *Gardnerella vaginalis*, a key pathogen in the development and recurrence of BV. The ratio of 80 kDa bLF to its 43 kDa and 37 kDa fragments remains high (> 80%) over at least the first 12 h of the time course while the tablet is in the Dissolution phase or early Washout phase. There is considerable inter-subject variability, as illustrated by the large 95% confidence interval error bars shown in Fig. 5b as well as the individual subject data shown from two additional subjects that also received the same formulation (Figs. 1 and 3). This variability relates to both the time of tablet dissolution and extent of proteolysis. For example, the subject shown in Fig. 1 had substantially more nicking than the subject in Fig. 3. The subject whose specimens are shown in Figs. 2 and 4 was a high proteolyzer, as VF specimens after administration of two different formulations both showed substantial amounts of the 43 kDa fragment even at early time points.

### Fragmentation patterns of bLF

**Fig. 8:**
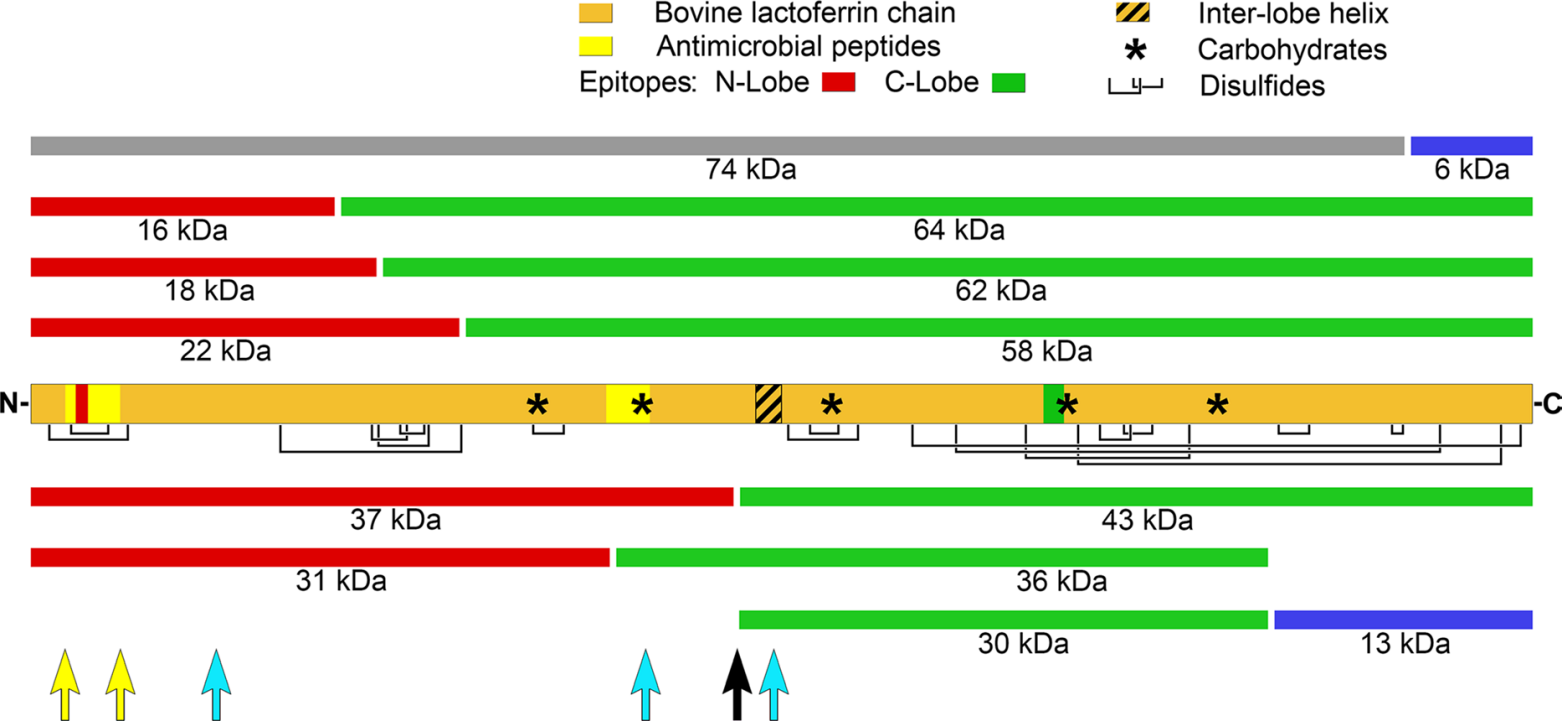
Fragmentation model for bLF in VF. The central orange bar represents intact 80 kDa bLF showing disulfide bonds (black lines below bar), carbohydrate moieties (asterisks), the inter-lobe helix (cross-hatched), mAb epitopes (N-lobe red and C-lobe green on the orange bar), and the antimicrobial peptides lactoferricin B (Tomita et al. 1991; yellow, left) and lactoferrampin (van der Kraan et al. 2004; yellow, middle). Bars above and below the central bar represent fragments identified in our previous studies (Hopp et al. 2022, from which this figure has been updated). Red bars possess the N-lobe epitope, while green bars possess the C-lobe epitope. The 74 kDa bar (gray) contains both epitopes, while the small blue bar fragments do not contain either epitope. Arrows indicate significant proteolytic cleavage sites reported for pepsin (yellow, Tomita et al. 1991; and this work), trypsin (blue, Rastogi et al. 2014a) and for VF in this report (black arrow)

Proteolysis of bLF generates a reproducible pattern of fragments in clinical VF samples, which we characterized in some detail previously (Hopp et al. 2022). Figures 1–4 show that, although this pattern is repeated from subject to subject and dose to dose, significant variations do occur. Figure 8 presents a schematic view of the 80 kDa bLF chain and its VF fragments, including key features useful in interpreting the discussion that follows.

### 80 kDa bLF

The PAGE gels in Figs. 1, 2, 3, 4 offer an information-rich starting point for analyzing bLF proteolysis by VF. Figure 1 shows a representative example of nicking of 80 kDa bLF. On the left, the non-reduced gel has dark bands for 80 kDa bLF starting at 8 h after dosing and persisting up to 24 h despite the rapid appearance of some proteolysis fragments. However, the righthand reduced gel shows that the apparent resistance to proteolysis of the 80 kDa material in the non-reduced gel is not the case when the same samples are analyzed under reduced conditions. The reduced gel demonstrates that the 80 kDa material was indeed attacked by protease(s) and, after disulfide bond reduction, a large variety of nicked species are revealed. These include bands of 74, 62/64, 58, kDa, in addition to the fragments commonly seen at 43, 37, 31, 22 kDa. This nicking phenomenon is most apparent at the 24 h time point, where a dark band at 80 kDa, non-reduced, is shown to have almost no intact 80 kDa material in the corresponding reduced lane. In Fig. 3, the same formulation was administered to a different subject and significantly less nicking was observed.

### 43 kDa fragment

The 43 and 37 kDa fragments represent the products of a single proteolytic cleavage of the 80 kDa molecule and both retain iron binding capacity (Hopp et al. 2022), albeit with undetermined affinities. While the 43 kDa fragment is often among the most prominent bands produced, it is subject to significant variability, especially by nicking (compare Figs. 1, 3.

### 37 kDa fragment

The counterpart of the 43 kDa fragment, this fragment usually appears simultaneously on PAGE gels in roughly equivalent amounts, as seen of Figs. 1, 2, and 4. This fragment often appears less densely stained than its 43 kDa counterpart. However, it is worth noting that it typically is the darkest component of a triplet of bands, 40, 37, and 35 kDa, seen most clearly in Fig. 4. The source of this weight polymorphism is not fully understood at present, but it is likely related to carbohydrate moiety variations reported in the literature (Wei et al. 2000). The N-lobe has two N-linked glycosylation sites at Asn 233 and Asn 281, the latter of which is commonly not glycosylated. The three bands seen on PAGE gels make a group consistent with the 37 kDa main band being as Wei described, that is, no glycosylation on Asn 281 but full glycosylation at Asn 233. Relative to this main form, the minor band at 35 kDa could represent non-glycosylation of both Asn 233 and Asn 281. The heavier 40 kDa band could be the doubly glycosylated form, with carbohydrate attached to both Asn 233 and Asn 281. At present, this hypothesis is not proven experimentally, but offers an explanation for the consistent appearance of these three bands as a co-varying triplet.

### 22 kDa fragment

While the 22 kDa fragment can be considered a sub-fragment of both the 37 kDa and 31 kDa N-lobe fragments, it is not true that there is a sequential breakdown from 80 to 73 to 31 to 22 kDa. Instead, as can be seen clearly on Figs. 1 and 2, the 22 kDa component arises as rapidly as both the 37 and 31 kDa fragments. This emphasizes that the initial proteolytic attack, nicking, and clipping to sub-fragments all occur simultaneously as random attacks by VF protease(s) on bLF.

### 18 kDa and 16 kDa fragments

The 18 and 16 kDa pair of bands are interesting because they often arise simultaneously, and often in equally dark bands, as in Figs. 1 and 2. Sometimes, their occurrence is not equivalent, and the 16 kDa appears in larger amounts or with different timing relative to 18 kDa (Fig. 2). Occasionally the 18 kDa component is entirely lacking while 16 kDa is strongly present, as in Fig. 4. Also, Fig. 2 shows 18 kDa disappearing with time, while 16 kDa remains. While it is tempting to speculate that 16 kDa is derived from 18 kDa by an additional nick that removes a 2 kDa piece, data for this are not available yet. In fact, the locations of either fragment within bLF’s structure remain imprecisely defined and the identities of the protease or proteases is unknown. Clarifying their precise locations and the proteolytic sites that produce them will add insight into the VF proteolytic process as well as the substrate specificity of the second protease.

### Additional products of proteolysis of bLF by human vaginal fluid

The molecular species discussed above do not offer a complete analysis of all fragments present in all clinical samples, rather they constitute a representative sample illustrating the commonalities and differences between fragments seen in different women and with different dose formulations. The N-terminal 37 kDa and C-terminal 43 kDa iron-binding fragments were the most prominent pair in most clinical samples, though as Figs. 1, 2, 3, 4 showed, they may exhibit substantial variation from subject to subject or timepoint to timepoint.

Other fragment pairs totaling 80 kDa that can be found in the gels herein include the two bands at 16 and 18 kDa which match a doublet band at 62 and 64 kDa (16 + 64 = 80 and 18 + 62 = 80), and a 22 kDa band matched with a 58 kDa band (22 + 58 = 80). A pair including a 31 kDa N-lobe plus a 49 kDa C-lobe fragment is less often seen. Notably, in some cases one fragment of a pair is more prominent than the other, suggesting sub-fragmentation of one of the pair while the other remains intact. We also previously noted a 74 kDa fragment possessing both the anti-N- and anti-C-lobe mAb epitopes, paired with a C-terminal 6 kDa non-epitope-bearing fragment (74 + 6 = 80). This pair was encountered again on several Coomassie gels presented here, especially Figs. 1, 2, and 4.

Additional nicking likely occurs within these bLF fragments. In several of the PAGE gels, the 43 kDa band is prominent when non-reduced but diminished when reduced. This is seen most clearly on the PAGE pairs in Figs. 1 and 3. In these cases the 43 kDa fragment must have carried one or more protease nicks within it, so that reduction of disulfides released smaller sub-fragments, which were hard to identify among the complex mixture of multiple bands in the 3-to-14 kDa region. The 37 kDa N-lobe fragment showed a similar nick-related behavior, seen most clearly in Fig. 1. Careful review of Figs. 1, 2, 3, 4 demonstrates that the amounts of other bands could be influenced by nicking as well (31 kDa; 22 kDa).

### Potential identities of the responsible proteases

Lactoferrin isolated from bovine milk includes a small percentage of proteolytically nicked molecules, attributed to trace amounts of proteases in milk, including plasmin, milk acid protease, and proteases derived from leukocytes (Kaminogawa et al. 1980; Politis et al. 1992; Aslam and Hurley 1997). These nicks primarily occur in the N-lobe, yielding fragments with molecular weights in the 30-to-40 kDa range for the nicked N-lobe and in the 40-to-50 kDa range for the corresponding C-lobe. The present work shows that VF proteases consistently nick bLF in this same region, but at a unique site. This major cleavage site, located at tyrosine 324 (Hopp et al. 2022) had not been reported in any previous study of bLF proteolysis.

Mass spectrometry analysis (Hopp et al. 2022) also found a second strong indication for cleavage after phenylalanine 569 to yield an N-terminus arginine 570, clipping the 43 kDa fragment into a 30 kDa fragment bearing the C-lobe epitope plus a 13 kDa C-terminus fragment with no epitope, shown in Fig. 8, lower right. Other N-termini were also detected, with N- and C-termini located throughout the entire sequence of bLF with no absolute pattern of preferred amino acid substrates, but with a slight preference for arginine C-termini or cleavage between large hydrophobic amino acids. However, sufficient ambiguity remained to preclude identifying the responsible protease(s) with certainty.

Recent proteomics analysis of VF demonstrated the presence of many proteases, especially serine- and cysteine-class enzymes as well as many kallikrein family members (Shaw et al. 2007; Muytjens et al. 2018). However, our ex vivo digests (Table 1, Fig. 7) were carried out at pH 3.3, so these enzymes would have been inactivated by acid. Therefore, other enzymes are likely involved in vaginal bLF proteolysis. For example, cathepsins D and E, members of the pepsin family of aspartyl proteases, have pH-activity profiles compatible with low pH, and specificities differing from pepsin (Arnold et al. 1997; Sun et al. 2013). These or other aspartyl proteases are strongly implicated by the inhibition seen when the pepsin inhibitor, pepstatin, was added to the reaction (Fig. 7). However, the incompleteness of the inhibition suggests that other members of the large aspartyl protease family may be involved. Several members of the Cys-cathepsin family of proteases are active at acidic pH and can cleave after arginines (Choe et al. 2006; Vidak et al. 2019). The occurrence of aromatic amino acids phenylalanine and tyrosine at P1 suggests the specificity of chymase or cathepsin G (Thorpe et al. 2018) although again, low pH would inhibit these serine proteases. Transmembrane serine proteases attached to vaginal epithelial cells have been reported, which possess strong preferences for arginine (Barré et al. 2014), but these would also be inhibited at low pH. In contrast, the seminal aspartyl proteinase, gastricsin, can be activated by the low pH of VF and remain active for hours (Szecsi and Lilja 1993). As mentioned, this enzyme shares target specificity with pepsin and seems unlikely to be acting on bLF because no lactoferricin B was detected. Also noted above (Fig. 7) the lack of an effect by adding EDTA rules out proteinases that utilize catalytic metal ions or are activated by divalent metal ions.

### Absence of lactoferricin B

We previously reported the absence of lactoferricin B or lactoferrampin (Hopp et al. 2022) among VF fragments of bLF measured by western blot. In the present work, our specific digestion reactions with pepsin, stomach fluid, and VF enabled direct comparisons on PAGE and western blots. Figure 6 definitively establishes that while lactoferricin B is readily produced by pepsin and stomach fluid, the comparable experiment with VF was just as clearly negative for the peptide. Bacterial vaginosis is a condition dominated by gram positive bacteria, thus the significance of the absence of lactoferricin B is unclear. However, its lack does not preclude potential antimicrobial activity among the other fragments observed here. More experimentation will be required to address this issue in the future.

### Persistence of iron binding protein species

The experiments presented in this paper demonstrate that, while the rates of proteolysis may vary between individuals, a sustained release of 80 kDa bLF typically occurs as newly dissolved bLF replaces degraded material. Consequently, both intact bLF and its large iron-binding fragments can persist for up to 24 h post-dose, especially the 43 kDa and 37 kDa iron binding fragments. In those subjects with more substantial proteolysis, these latter fragments probably compensate for loss of the 80 kDa molecule, so the VF continues to have significant levels of iron-binding protein. This implies that substantial iron sequestering activity should be available even after the 80 kDa molecule has been cleaved. This iron sequestering activity would be expected to alter the microbiome by favoring normal, iron-independent lactobacilli species over iron-requiring BV pathogens like *Gardnerella vaginalis*.

## Conclusions

This report describes dissolution, proteolysis, and washout of vaginally administered bLF. Large amounts of 80 kDa intact bLF persisted in VF for up to 24 h, despite limited proteolysis, indicating that proteolysis was not the major clearance mechanism for vaginally administered bLF. The 80 kDa molecule was initially cleaved into 37-kDa N-lobe and 43-kDa C-lobe fragments. Slow dissolution of the MTbLF tablet resulted in the persistence of the 80, 43, and 37-kDa bLF species in VF throughout the Dissolution and Washout phases of the suppository. The identities of the bLF-degrading VF protease(s) were explored, and evidence was gathered from inhibition studies that an aspartyl-protease family member was involved. Nevertheless, distinct patterns of cleavage fragments, and the complete absence of the pepsin cleavage product lactoferricin B demonstrated a lack of pepsin-like activity in VF. Additional experimentation will be required to elucidate the exact nature of the protease(s) involved. The results presented here demonstrate persistence of iron-binding polypeptides of bLF in VF, supportive of a once-daily dosing regimen. The sustained iron-binding capacity provides a therapeutic rationale for a nutritional immunity approach to treating BV and other vaginal conditions by maintaining an iron-depleted environment favoring lactobacilli over pathogenic species.
